# LogLoss-BERAF: An ensemble-based machine learning model for constructing highly accurate diagnostic sets of methylation sites accounting for heterogeneity in prostate cancer

**DOI:** 10.1371/journal.pone.0204371

**Published:** 2018-11-02

**Authors:** K. Babalyan, R. Sultanov, E. Generozov, E. Sharova, E. Kostryukova, A. Larin, A. Kanygina, V. Govorun, G. Arapidi

**Affiliations:** 1 Federal Research and Clinical Center of Physical-Chemical Medicine of Federal Medical Biological Agency, Moscow, Russian Federation; 2 Moscow Institute of Physics and Technology (State University), Dolgoprudny, Moscow Region, Russian Federation; 3 Shemyakin-Ovchinnikov Institute of Bioorganic Chemistry of the Russian Academy of Sciences, Moscow, Russian Federation; Stockholm University, SWEDEN

## Abstract

Although modern methods of whole genome DNA methylation analysis have a wide range of applications, they are not suitable for clinical diagnostics due to their high cost and complexity and due to the large amount of sample DNA required for the analysis. Therefore, it is crucial to be able to identify a relatively small number of methylation sites that provide high precision and sensitivity for the diagnosis of pathological states. We propose an algorithm for constructing limited subsamples from high-dimensional data to form diagnostic panels. We have developed a tool that utilizes different methods of selection to find an optimal, minimum necessary combination of factors using cross-entropy loss metrics (LogLoss) to identify a subset of methylation sites. We show that the algorithm can work effectively with different genome methylation patterns using ensemble-based machine learning methods. Algorithm efficiency, precision and robustness were evaluated using five genome-wide DNA methylation datasets (totaling 626 samples), and each dataset was classified into tumor and non-tumor samples. The algorithm produced an AUC of 0.97 (95% CI: 0.94–0.99, 9 sites) for prostate adenocarcinoma and an AUC of 1.0 (from 2 to 6 sites) for urothelial bladder carcinoma, two types of kidney carcinoma and colorectal carcinoma. For prostate adenocarcinoma we showed that identified differential variability methylation patterns distinguish cluster of samples with higher recurrence rate (hazard ratio for recurrence = 0.48, 95% CI: 0.05–0.92; log-rank test, p-value < 0.03). We also identified several clusters of correlated interchangeable methylation sites that can be used for the elaboration of biological interpretation of the resulting models and for further selection of the sites most suitable for designing diagnostic panels. LogLoss-BERAF is implemented as a standalone python code and open-source code is freely available from https://github.com/bioinformatics-IBCH/logloss-beraf along with the models described in this article.

## Introduction

Prostate cancer (PC) is one of the most frequently diagnosed oncological diseases in males worldwide [1]. Like most other cancers, the early stages of PC are characterized by an asymptomatic course, which substantially impedes its early diagnosis [2]. Advances in the past decade of research, particularly in genetic studies, have provided a deeper understanding of the molecular mechanisms underlying PC pathogenesis, and these advances can serve as the basis for the development of effective molecular genetic methods for early diagnosis of this disease [3].

The latest experimental data have clarified the role of genetic and epigenetic factors in PC pathogenesis [4]. Among these factors, epigenetic alterations, particularly aberrant DNA methylation of CpG dinucleotides in genes, are of special interest. These alterations are often functionally related to the expression regulation of tumor suppressors and oncogenes at early stages of both prostate cancer and other types of oncological diseases [5,6].

Despite the advantages of this approach, the application of such epigenetic markers in diagnostic practice tends to have certain limitations, mostly at the technical level. Among the most widely used are whole-genome DNA methylation analyses based on either high-throughput sequencing or DNA hybridization arrays. For example, the Infinium HumanMethylation450 BeadChip array (HM450) can be used to estimate methylation levels for 98,9% of all characterized genes (according to the UCSC RefGenes database) [7]. However, such methods are not always suitable for routine laboratory diagnostics due to their high cost and complexity compared to PCR-based methods and due to the large amount of sample DNA required for the analysis. Quantitative methylation-specific PCR techniques, such as methylation-sensitive high-resolution melting (MS-HRM), which requires only 10 ng of DNA, are more convenient for clinical pathology analysis, and their development may allow clinicians to switch to less invasive diagnostic methods in the future [8]. However, despite their relative technological simplicity, these methods are not designed to analyze large numbers of markers simultaneously. Thus, the number of candidate CpG sites often must be restricted, which results in decreased sensitivity and specificity of the test.

High molecular heterogeneity of tumors compared to non-tumor tissues, which includes DNA methylation patterns, presents another challenge for clinical diagnostics. Significant DNA methylation variability in tumors is common in prostate cancer and has been shown to be the case for many other oncological diseases [9]. The existence of different molecular tumor subtypes makes it difficult, and sometimes even impossible, to select informative and reproducible diagnostic signatures, and this is the reason why the results of conventional classification methods for marker selection from limited datasets are often irreproducible with independent data [10]. The simplest method of forming a marker panel is the selection of top N differentially methylated sites. The major problem of this approach is the possibility of redundant markers being included in the model due to the fact that the selection of each next marker is independent of the markers already present in the model. Moreover, this method does not allow to identify markers that are specific only for a small cluster of samples belonging to a certain heterogeneity subgroup because such markers have insufficient level of differential methylation. Many experimental studies suggest that methylation site selection methods, including both the estimation of the average difference in methylation levels and calculation of the differential methylation variability of different sites, may prove to be more reliable [11]. Thus, there is a need for an approach that would take into account the high variability of the source dataset of analyzed candidate markers and produce a limited number of final markers.

Currently used feature selection methods can be divided into three main categories [12]: embed, wrapper, and filtering methods. Embed methods are characterized by joint optimization of the classifier, model construction and feature subset selection. The main approach here is regularization, which is implemented in well-known and widely used algorithms such as LASSO [13]. Wrapper methods include initial training on different factor subsets, and the final model is defined by optimization of a previously selected metric. These methods use forward selection (the algorithm starts from an empty set, and new factors are iteratively added to it) and backwards selection (the algorithm iteratively removes “odd” factors from the set) [12]. This method reconstructs interactions between factors more effectively but at the same time risks overfitting when a dataset contains few samples and many factors. Finally, filtering methods are based on statistical tests and usually process factors separately to calculate their correlation with the goal variable. These methods tend to be faster than others, but they do not consider interactions between factors.

Many studies [14–22] have focused on feature selection analyses for gene expression and mutational data, but there are few studies describing similar approaches to methylation data. Alkuhlani et al. suggested using a combination of feature selection through Fisher’s test and t-tests, a genetic algorithm with SVM-RFE as an optimizable function, and an SVM classifier [23]. Ma Z et al. used a variational Bayes beta mixture model as a method for selection and optimization of prognostic markers [24]. However, neither of these models supports initial restriction of the factor subset size, which makes the resulting sets hard to translate into routine laboratory practice.

The aim of this study was to develop a framework for selection of a limited number of diagnostically informative DNA methylation sites and to estimate its potential diagnostic efficiency. We evaluated the method using publicly available whole-genome DNA methylation data for prostate cancer, as one of the highly heterogeneous cancers, and for several other oncological diseases.

## Materials and methods

### Datasets

DNA methylation data used in this study were acquired using lllumina Infinium HumanMethylation 450k BeadChip technology [25]. The development of the model and the estimation of its parameters were performed with the use of DNA methylation data from the TCGA PRAD project. We used DNA methylation data from tumor and corresponding non-tumor (morphologically unchanged) prostate tissue samples. We applied the framework to other types of oncological diseases to demonstrate its efficiency. Since one of the promising current trends is non-invasive PC diagnostics based on DNA methylation markers obtained from urine samples [26,27], we also analyzed methylation data for urothelial bladder carcinoma (BLCA), kidney renal clear cell carcinoma (KIRC), and kidney renal papillary cell carcinoma (KIRP). As PC is often co-localized with colon adenocarcinoma, we also applied the framework to TCGA COAD data. The samples used for the framework development and validation are listed in Table 1.

**Table 1:** List of tumor (T) and non-tumor (N) samples and datasets used for model training and validation.

Source	Training Set	Test Set	Total
**Prostate cancer**			
**FRCC PCM FMBA**	8T, 11N	13T, 16N	48
**TCGA (PRAD)**	117T, 15N	176T, 23N	331
**GSE55479**	0	143T, 0N	143
**GSE38240**	0	8T, 4N	12
**GSE73549**	0	77T, 15N	92
**Bladder cancer**			
**TCGA (BLCA)**	134T, 9N	201T, 14N	490
**Colorectal cancer**			
**TCGA (COAD)**	133T, 15N	200T, 22N	370
**FRCC PCM FMBA**	6T, 9N	8T, 11N	34
**Kidney cancer (KIRC)**			
**TCGA (KIRC)**	116T, 52N	174T, 78N	420
**Kidney cancer (KIRP)**			
**TCGA (KIRP)**	101T, 63N	151T, 104N	419

#### Prostate adenocarcinoma

FRCC PCM dataset: our own dataset for 48 samples (GSE74013) is available at Gene Expression Omnibus (GEO; www.ncbi.nlm.nig.gov/geo/); TCGA PRAD dataset: data for 331 samples obtained from The Cancer Genome Atlas (https://tcga-data.nci.nih.gov/tcga/, TCGA PRAD). The samples were selected according to age and clinical criteria (a detailed description is provided in S1 Table); PRAD datasets downloaded from Gene Expression Omnibus: GSE55479 (143 samples), GSE38240 (12 samples) and GSE73549 (92 samples).

#### Urothelial bladder carcinoma

TCGA BLCA dataset: 351 samples.

#### Kidney cancer

Renal clear cell carcinoma–TCGA KIRC dataset: 420 samples; Kidney renal papillary cell carcinoma–TCGA-KIRP dataset: 419 samples.

#### Colon adenocarcinoma

FRCC PCM dataset: our own data for 34 samples (GSE42752); TCGA COAD dataset: 370 samples.

#### White blood cells

For additional validation of the model, we used DNA methylation data from 200 leukocyte blood fraction samples obtained from individuals of different ages (GSE87571).

### Preprocessing

Preprocessing of raw IDAT files was performed with the RnBeads package [28]. Systematic batch effect correction was done using the ComBat algorithm from the sva package [29]. Normalization and background correction were performed with NOOB [30] and BMIQ [31] algorithms, which demonstrated the best results corrected for technical errors when used in combination [32].

### Combined feature selection

The methylation level of each CpG site is represented as a beta-value, β, which is calculated as follows [25]:

$$\beta =\frac{M}{M+U+100}$$ (1)

where M is the methylated intensity and U is the unmethylated intensity of each probe.

Henceforth, we will refer to a vector of beta values β ∈ (0, 1)^N^, where N is the number of samples, as a feature. Further feature selection is based on the following biological and technically required rules and limitations:

A selected feature set (henceforth called a signature) can include heterogeneously methylated CpG sites.

A selected signature must include not more than a predefined number of CpG sites (factors).

Methylation values of CpG sites included in the signature must differ between the analyzed classes (i.e., pathology vs. non-pathology) by more than a predefined value and may vary within the experimental level of accuracy.

The feature selection method must be applicable to unbalanced sets, where the number of the samples from one class (i.e., pathology) is much greater than that of another or where the number of factors is much greater than the total number of samples.

**Fig 1:**
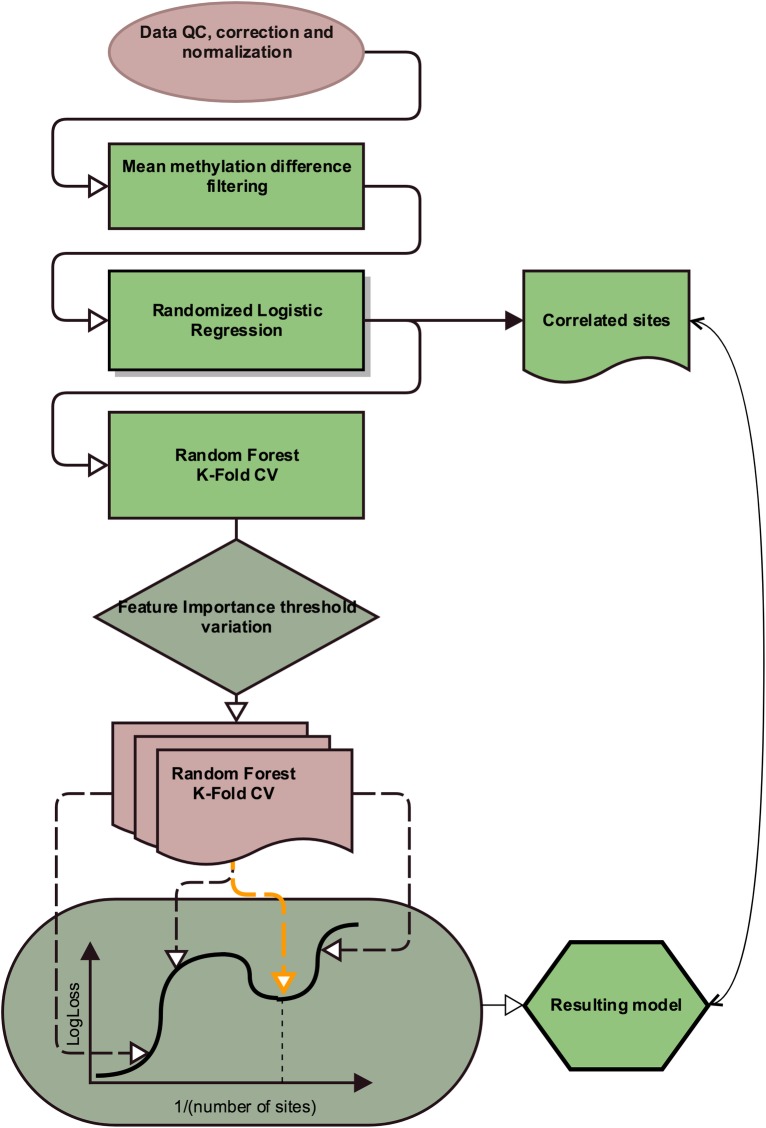
Pipeline of the proposed method.

The scheme of the model construction algorithm is shown in Fig 1. Let P be the upper limit for the number of features in the diagnostic panel, C—number of analyzed classes, Δβ—minimum difference between average methylation levels. The first step consists of the selection of the factors for which the average methylation between at least one pair of groups differs by more than the user-defined value Δβ (Eq 2):

$$\exists \;i,j\in \{1,\ldots ,C\}:\;abs\left(mean(\beta_{i}^{n})-mean(\beta_{j}^{m})\right)>\Delta \beta$$ (2)

where $\beta_{i}^{k}$ represent methylation beta values in a sample subset k belonging to class i.

The next step involves a combination of two feature selection methods. The first one is randomized logistic regression (RLR), also known as the stability method [33], implemented in the scikit-learn 0.17.1 package [34]. In RLR, the original set is split randomly, and the sites that have non-zero coefficients after regularization are selected from the resulting subsets. The RLR method was selected because it allows selection of a limited number of the most significant sites for classification using L1 regularization and because it can identify highly correlated sites and include them into the resulting set due to random splitting at each iteration of randomization. Additionally, site selection on a subset of samples can potentially account for heterogeneity among samples. Nevertheless, because of the stochasticity of the process, not all highly correlated features may be included in the resulting set, as some of them may be discarded during model construction as non-informative compared to those already included in the model. However, these features can be as valuable as the selected ones; thus, we perform a pairwise correlational analysis in order to avoid data loss.

In the following steps, we used a random forest (RF) algorithm. RF is a popular and efficient method for classification problems and is based on ensembles of decision trees and bootstrap aggregating, which is designed to avoid overfitting [35]. To handle unbalanced data, sample weights are adjusted inversely proportional to class frequencies.

A trained classifier provides the estimate of the importance factor used for sample classification, which can be used for further feature selection.

The feature importance threshold is then varied to construct a random forest for each factor subset, and 10-fold cross-validation is performed. This approach is a popular machine learning method that provides an unbiased estimate of model accuracy [36]. Classification efficiency is estimated based on several metrics: precision, recall and LogLoss, also called cross-entropy loss or logistic regression loss [37]. The latter represents a classifier accuracy estimate and allows the prediction of classes themselves (y_ij_) and their probabilities (p_ij_). (Eq 3)

$$LogLoss=-\frac{1}{N}\sum_{i=1}^{N}\sum_{j=1}^{C}y_{ij}*logp_{ij}$$ (3)

The usage of LogLoss metrics is motivated by the fact that we want to impose penalties both for false predictions and for low confidence in true ones. This approach allows us to identify less noisy sites.

Next, when model quality values for different feature subsets have been obtained, a local minimum search for the LogLoss function of the number of factors is performed in the neighborhood of the desired number of sites. The resulting set of factors is selected as a set of minimum size such that its logistic regression loss value differs from that of the local minimum by not more than one standard error. (Eq 4)

$$\min_{x\in (1,P)}x:|LogLoss(x)-\min_{x_{l}\in (1,P)}\;LogLoss(x_{l})|<1\;s.e.$$ (4)

One of the advantages of LogLoss usage is the ability to detect outliers. For example, if some samples in the input data are assigned to a wrong class or differ substantially from others, the resulting model will be strongly penalized for their use, and the loss function value itself will be relatively high. Class membership predictions and input object class data are incorporated to construct a list of potential outliers by marking objects for which the probability of belonging to a wrong class is above the threshold.

The resulting classification performance was evaluated using AUC metrics.

## Results and discussion

The developed framework was applied to the datasets listed in Table 1 with the following parameters: user-defined minimum variation of the average methylation levels between classes Δβ ≥ 0.2; 1500 RLR iterations, a random forest of 500 trees for factor importance estimation, and an intermediate random forest consisting of 1000 trees parameters were obtained through nested cross-validation.

### Model construction for prostate cancer

To construct a diagnostic model for prostate adenocarcinoma, we analyzed 626 samples: the training set contained 151 samples, and the independent test set contained 475 samples. The resulting model produced by LogLoss-BERAF included data from 9 methylation sites (Table 2, S2 Table) that showed high levels of differential methylation between the groups (S1 Fig); the model demonstrated 0.95 recall, 0.95 precision, a 0.95 F1-score and 0.97 AUC (95% CI: 0.94–0.99) on the test set.

**Table 2:** Prostate adenocarcinoma classification model sites with their positions and their corresponding gene name and group.

IlmnID	Chr	Position	Gene name	Group
Prostate adenocarcinoma				
cg02361803	chr1	2014371	PRKCZ	Body
cg11448068	chr2	191045026	C2orf88	TSS1500
cg16100120	chr2	56150475	EFEMP1	TSS200
cg00817367	chr12	52401214	GRASP	Body
cg18844382	chr14	23834977	EFS	TSS200
cg00402172	chr16	68118754	NFATC3	TSS1500
cg14621217	chr17	80944134	B3GNTL1	Body
cg16849024	chr19	41934210	B3GNT8	5'UTR
cg22059073	chr22	17602570	CECR6	TSS1500

### Algorithm allows to construct high efficiency panels of biomarkers without a priori knowledge of their diagnostic efficacy

Prostate cancer analysis provides a good opportunity for the optimization of marker selection methods because epigenetic alterations are highly prevalent and arise early in prostate tumorigenesis. The most recent studies have identified many DNA methylation alterations as potential biomarkers for prostate cancer diagnostics. Feature selection was generally carried out based on a prioritized list of genes showing the most significant differences in methylation levels between tumor and non-tumor sample groups.

*GSTP1* is the most well-characterized epigenetic biomarker for PC. DNA methylation of *GSTP1* is present in almost all PC cells but is absent or present in low levels in normal cells. However, the estimation of *GSTP1* methylation levels does not demonstrate high specificity and recall (0.88 and 0.91 respectively) of *GSTP1* as a diagnostic biomarker [38–40]. This issue could be addressed in part using multigene promoter methylation testing. For the moment, good clinical utility method of estimating methylation levels has been shown for promoters of *GSTP1*, *APC* and *RASSF1* genes [41]. This model may be used to predict negative histopathological results in repeat prostate biopsies.

To assess the diagnostic performance of the developed model, we compared its results to the precision and recall values calculated for the 3-gene model described by Stewart et al. (2013) and several other published multigene models. We reproduced the calculations of these models and applied them to the datasets being analyzed in this study. The 3-gene model based on combined analysis of average methylation levels for *GSTP1*, *RASSF1* and *APC* demonstrated good results when applied to our data: 0.92 AUC (95% CI: 0.87–0.96), 0.91 precision, 0.89 recall, and 0.89 F1-score, which nonetheless represents a lower performance compared to our model.

Chung et al. demonstrated the diagnostic significance of *SPOCK2* and *NSE1* gene methylation with 0.80 recall and 0.95 precision (AUC was not reported) [42]. The proposed logistic regression model applied to our data had 0.90 precision, 0.87 recall and an 0.88 F1-score with 0.91 AUC (95% CI: 0.86–0.96).

One of the most common approaches is to first calculate differential methylation for individual sites and then select statistically significant differences to construct a model. We applied this method to select 3 methylation sites (*cg00054525*, *cg16794576* and *cg24581650*) using a linear mixed model [43] and then used logistic regression to construct a diagnostic model that had 0.845 recall and 0.917 specificity, with the resulting AUC of 0.92 for the test set. When trained and tested on our datasets, the model demonstrated 0.93 AUC (95% CI: 0.88–0.95), 0.92 precision, 0.92 recall, and a 0.91 F1-score.

Tumor-associated events at the DNA methylation level can vary greatly in scale in prostate cancer, and therefore, it is reasonable to consider a signature that uses more than 3 factors; their selection can be conducted independently without an initial rating of all sites and extraction of a top subset. For example, Tang et al. [44] considered 8 hypermethylated sites (*cg06363129*, *cg08843517*, *cg03576469*, *cg05385513*, *cg07220448*, *cg11417025*, *cg20883831* and *cg23824801*) located in promoter regions. The models constructed using logistic regression had from 0.91 to 0.94 AUC for individual methylation sites and 0.94 AUC when a combination of sites was used (recall and precision values were not reported). The 8-site model reproduced on our datasets demonstrated 0.95 AUC (95% CI: 0.90–0.98), 0.93 recall, 0.92 precision, and a 0.93 F1-score. All obtained values are listed in S3 Table.

Thus, ensemble methods for model construction demonstrate higher efficiency than the identification of individual sites due to their capability to reveal various connections between factors and predicted classes, which provides more stable and reproducible results. Additionally, in contrast to supervised approaches to marker selection that impose tight restrictions on the candidate sites in terms of their methylation levels, variability, and differences between the groups or gene information, our unsupervised method has demonstrated high classification performance, both absolute and in comparison, with other prostate cancer diagnostic panels. This framework has also allowed us to build a small-sized signature and expand the list of known potential prostate cancer biomarkers.

### Highly correlated model sites can be interchanged without a loss of diagnostic significance

Highly variable data, such as DNA methylation profiles in oncological diseases, are often characterized by the presence of several factor subsets that show equal or close classification efficacy [45,46]. In our model, many highly correlated sites can be dismissed at the construction step because they do not carry new information compared to those already included in the model. However, such correlated sites can be as informative as individual sites from the model (S4 Table). We aimed to assess how replacing the sites from the resulting model with highly correlated sites could affect the classification efficacy. Sites were considered highly correlated if their Pearson correlation coefficient was greater than 0.85.

We performed 10,000 permutations where each site from the model could be replaced by one of the correlated sites. The resulting classification efficacy had an AUC value of 0.93 (95% CI: 0.90–0.97). Thus, certain model sites could be substituted with ones more convenient for practical use, i.e., considering region mappability or applicability for primer design.

### Method for model construction showed relatively high stability

In addition to accuracy, another important characteristic of an algorithm is its stability, which is crucial for tasks involving few samples and high dimensionality. Algorithm stability is defined as the variability of factor selection resulting from minor changes in the training set. For k sub-samplings from the initial set, the final stability is estimated as the average agreement over all subsampling pairs. Let *f*_*i*_ be the i-th subsampling, then the agreement can be calculated as Kuncheva index [47]

$$S=\frac{2\sum_{i=1}^{k}\sum_{j=i+1}^{k}\frac{rN-s^{2}}{s(N-s)}}{k(k-1)}$$ (5)

where N is the initial number of factors, r = |*f*_*i*_⋂*f*_*j*_| is the number of identical elements in the subsamplings, s = |*f*_*i*_| = |*f*_*j*_|.

Due to the identified characteristics of correlated sites, factors combined with correlated ones were used as *f*_*i*_. Our experiment included 100 bootstrapping iterations with 90% of samples randomly selected at each iteration and resulted in a relatively high Kuncheva index of 0.72, while LASSO alone obtained score of 0.55.

### The developed framework allows methylation sites that are highly heterogeneous between groups to be included in the resulting model

**Fig 2:**
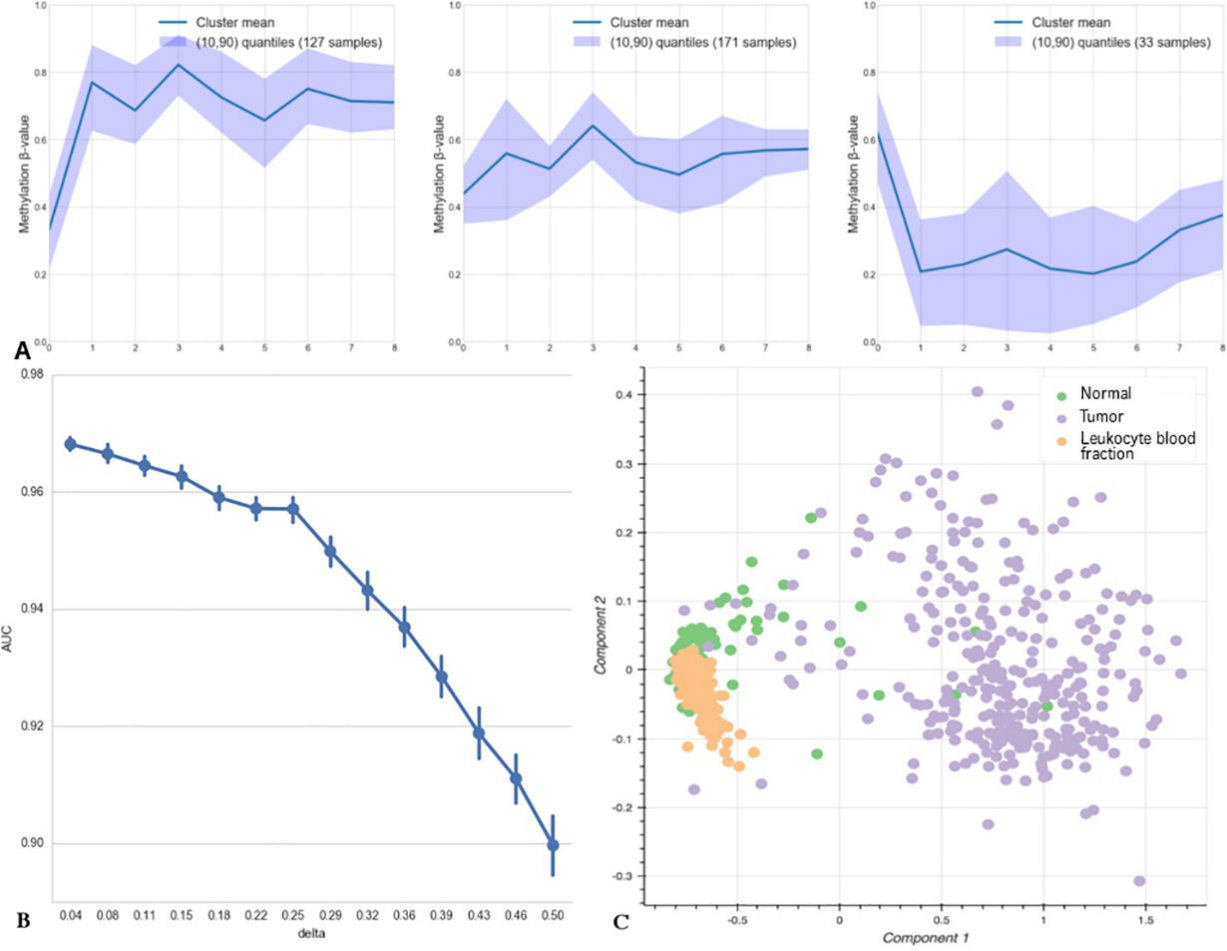
**A.** Clustering of tumor samples by shared methylation patterns of the sites included in the prostate cancer classification model. The X axis shows indices of model sites (0: cg02361803; 1: cg16100120; 2: cg11448068; 3: cg00817367; 4: cg18844382; 5: cg00402172; 6: cg14621217; 7: cg16849024; and 8: cg22059073). **B.** AUC changes depending on the noise level, delta, introduced into methylation levels of the sites included in the prostate cancer classification model. **C.** PCA graph for the sites of the PRAD diagnostic model with methylation groups assigned according to the data for leukocyte blood fraction from nominally healthy people.

The key point of the algorithm is its capability of efficient sample classification regarding the differential variability of the sites used in the model. In contrast, with supervised approaches based on strict selection of candidate markers by differential methylation level, our method allows for certain intra-group methylation level variability of candidate markers by differential methylation level, our method allows for certain intra-group methylation level variability of individual sites. For example, the methylation pattern of resulting model CpG sites in cancer samples allows them to be split into at least three main clusters (Fig 2A) using a k-means algorithm (skilearn v. 0.17.1). Different methylation patterns of samples combined with predictive values of the sites can be used for biological interpretation of metagenetic manifestation of the disease heterogeneity. Nonetheless, the clustering results produced by our model do not identify the same subtypes as those obtained by methylation analysis of the TCGA prostate cancer dataset and reported in The Cancer Genome Atlas Research Network study [48].

**Fig 3:**
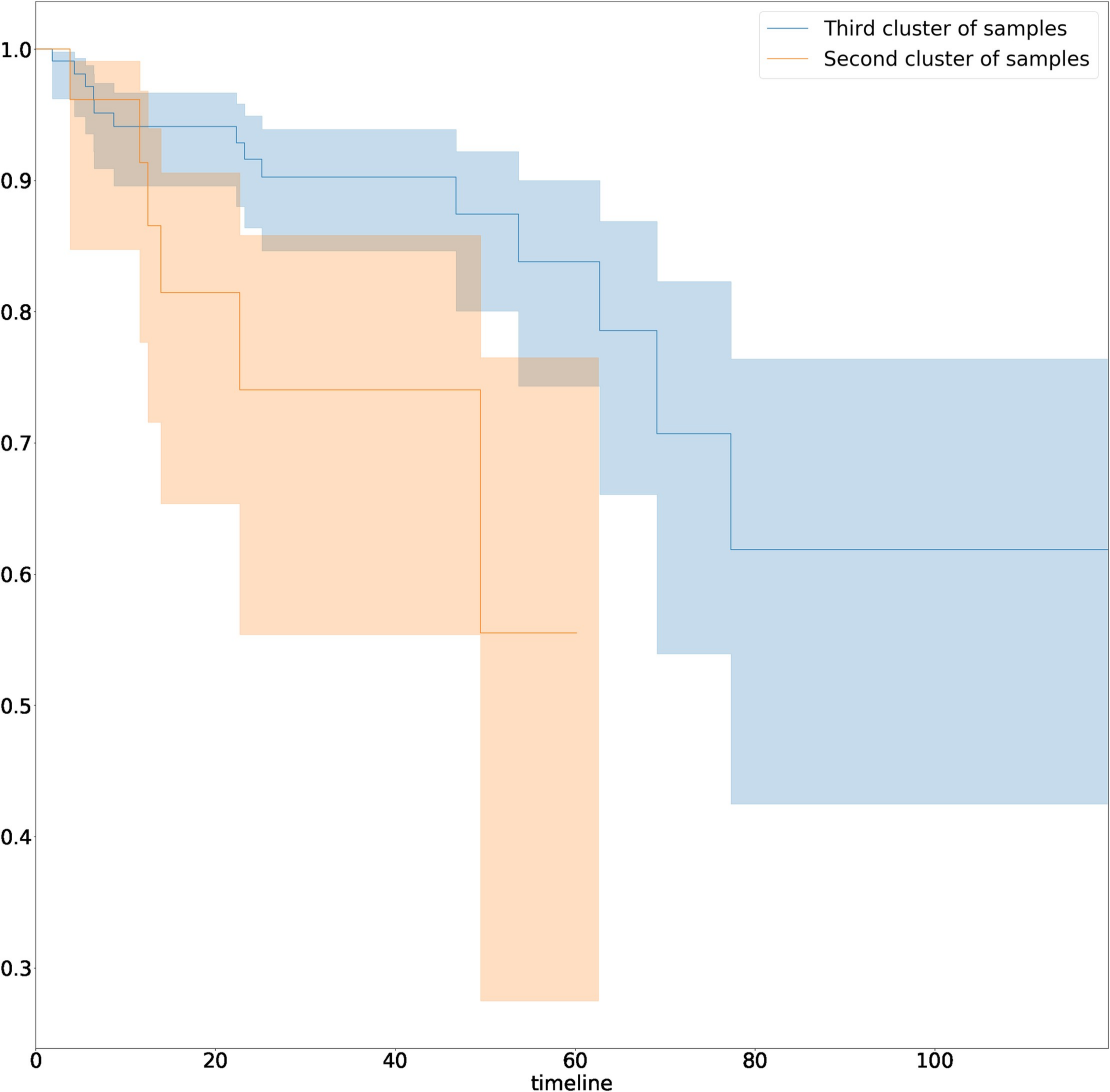
Kaplan-Meier curves for samples from second and third clusters. Clusters introduced in Fig 2A.

Nevertheless, the survival analysis of disease recurrence for samples from different clusters demonstrated statistically significant difference between clusters 2 and 3 (hazard ratio for recurrence = 0.48, 95% CI: 0.05–0.92; log-rank test, p-value < 0.03) (Fig 3), which indicates additional potential clinical applicability.

### The proposed method demonstrates tolerance to input data errors

One of the problems that arise in practical application of a machine learning model is its tolerance to noise in the input data, which occurs because of a batch effect, technical errors and other errors [49]. To assess our model, we gradually introduced noise Δμ_i_ into the methylation levels of the input data. Δμ_i_ values were varied randomly in the range of (−Δμ_i_; +Δμ_i_), the AUC was calculated for each Δμ_i_, and then, Δμ_i_ was increased (Fig 2B). In total, 1000 iterations were performed to estimate AUC variance at each step. The model demonstrated robustness at noise levels approximately 0.16 of the methylation *β*-value, which represents a good performance and suggests an opportunity for further development of the model into a sufficiently fast, inexpensive, robust and widely available method that will be useful for routine clinical diagnostics [50,51].

This model has also produced high AUC values on sufficiently noisy data (±0.5 *β*-value). We assumed that this result was due to the prevalence of tumor samples in the dataset and the associated high dispersion of methylation values compared to non-tumor samples. To check this hypothesis, we tested the model for type I errors by applying it to leukocyte blood fraction methylation data from 200 samples obtained from nominally healthy people. The PCA graph is shown in Fig 2C. The model classified all samples as non-tumors, which confirms the hypothesis that due to the high intragroup heterogeneity of tumor samples, the model tends to classify samples with highly non-uniform methylation levels as tumors. This finding also demonstrates the effective performance of the model considering the considerable discrepancy between the sizes of the datasets being analyzed.

### Biological function of genes containing model methylation sites and correlated sites

The present study is primarily concerned with methodical aspects of the development of bioinformatics approaches to the selection of candidate diagnostically informative DNA methylation sites. Since many highly correlated sites are excluded from the model during selection and since the resulting number of model sites is limited, the remaining model sites may not always represent the actual biological mechanisms associated with the disease. Despite this fact, we analyzed the possible functional role of the alterations in the genes included in the final model (Table 2). For most of these genes, the available information suggests that they may play a role in the pathogenesis of oncological diseases.

For example, *PRKCZ* codes for the isoform of protein kinase C involved in a variety of cellular processes, such as proliferation, differentiation and secretion. This gene is best known as being responsible for insulin-stimulated glucose transport. Cornford et al. were the first to show that the protein kinase C gene (PKC)-zeta (*PRKCZ*) mediates the malignant phenotype of human prostate cancer [52]. Recently, a splice variant of *PRKCZ* has also been shown to be a novel biomarker of human prostate cancer [53]. *PRKCZ* has also been characterized as one of four genes with higher autoantibody titers in PC and is considered a novel potential serological prostate cancer biomarker [54]. The role of *PRKCZ* methylation in the pathogenesis of type 2 diabetes [55] and its association with sunlight exposure in North Americans [56] are being discussed, but to date, there is no evidence of the contribution of *PRKCZ* methylation to prostate cancer pathogenesis.

*EFEMP1* was previously characterized as a biomarker for prostate cancer, for which epigenetic alteration occurs early in prostate carcinogenesis and, in association with histone deacetylation, progressively leads to gene down-regulation, fostering cell proliferation, invasion and evasion of apoptosis [57].

Decreased expression of the Fyn-associated substrate (*EFS*) gene involved in cell attachment is often associated with systemic recurrence of prostate cancer [58]. The association between *EFS* methylation and a considerable decrease in expression level in prostate cancer has also been observed [59]. The authors suppose that high *EFS* expression is important to suppress the malignant behavior of prostate cancer cells.

Many large-scale studies have reported an association between PC and methylation alterations in the *GRASP* gene coding for a general receptor for phosphoinositide-1-associated scaffold protein [60]. Further research concerning histologically benign prostate biopsy cores from cancer patients suggests that this marker is more likely to be methylated in histologically detectable cancer and may represent later events [61].

The *NFATC3* (nuclear factor of activated T-cells, cytoplasmic 3) gene plays a role in the regulation of gene expression in T cells and immature thymocytes. This gene is a member of the Wnt pathway and is associated with an increased risk of disease progression independent of clinical parameters among 7 other loci in an epithelial ovarian cancer model. Increased methylation at *NFATC3* is correlated with a poor response [62].

Although there is no solid evidence of association between C2orf88 methylation and prostate cancer, a study of colorectal cancer via integrative epigenomics and genomic data reported *C2orf88* to be among the 10 most significant differentially downregulated genes [63].

Therefore, we conclude that CpG sites included in the model lie within genes that have already been shown to contribute to the pathogenesis of PC or other types of oncological diseases.

### Framework application for other cancers

The suggested framework for the selection of diagnostically informative methylation sites can also be used for oncological diseases other than prostate cancer. To estimate LogLoss-BERAF performance for other types of cancer, we applied it to available DNA methylation data for kidney, bladder and colorectal cancer. The choice of urological cancer was determined by the fact that these cancers are often characterized by the presence of tumor cells in urine. As analysis of urine samples is one of the promising non-invasive methods for PC diagnostics, such a test would allow an additional specificity test of the prostate cancer model applied for the differential diagnosis of other urological cancers. The choice of colorectal cancer data is in turn explained by its co-localization with PC. Although patients with synchronous carcinoma of the bladder and colon or rectum are rare, there is a possibility of sample contamination with colon cellular material, including malignantly transformed cells, during a transrectal biopsy.

LogLoss-BERAF was applied to the available datasets (Table 1) for the listed cancers to select model and correlated methylation sites, and their diagnostic efficacy was estimated (Tables 3 and 4, S5–S8 Tables).

**Table 3:** Model classification efficacy metrics: precision, recall, F1-score and AUC for test sets and the number of sites per model obtained using LogLoss-BERAF for different types of oncological diseases.

Cancer type	Sites num.	Precision	Recall	F1 score	AUC
**Prostate Cancer**	9	0.95	0.95	0.95	0.97
**Colorectal Cancer**	3	1.0	1.0	1.0	1.0
**Bladder Cancer**	6	0.98	0.98	0.98	1.0
**Kidney Cancer (KIRP)**	5	0.98	0.98	0.98	1.0
**Kidney Cancer (KIRC)**	2	0.99	0.99	0.99	1.0

**Table 4:** Co-localized and diagnostically similar cancers: classification of model sites with their positions and corresponding gene names and groups.

IlmnID	Chr	Position	Gene name	Group
**Colon Adenocarcinoma**				
cg01588438	chr8	67344553	ADHFE1	TSS200
cg04456219	chr7	17274337	-	-
cg09287864	chr7	17274056	-	-
**Urothelial Bladder Carcinoma**				
cg06830167	chr1	7600135	CAMTA1	Body
cg10671066	chr1	160492861	SLAMF6	Body
cg14357535	chr2	25389040	POMC	5'UTR
cg03487935	chr7	51925284	-	-
cg17202717	chr7	1708823	-	-
cg01090433	chr16	82673506	CDH13	Body
**Kidney Renal Clear Cell Carcinoma**				
cg22274117	chr6	16713613	ATXN1	5'UTR
cg00347746	chr19	48970082	-	-
**Kidney Renal Papillary Cell Carcinoma**				
cg04951371	chr2	3317860	TSSC1	Body
cg22274117	chr6	16713613	ATXN1	5'UTR
cg13458609	chr9	130608923	ENG	Body
cg02921122	chr10	126712074	CTBP2	Body
cg02766539	chr17	57861641	TMEM49	Body

Because of the small number of non-tumor samples in the urothelial bladder carcinoma dataset, 167 non-tumor samples from the kidney cancer dataset were added to the dataset before splitting into train and test subsets. The usage of methylation data from non-malignant tissues of other organs of the urogenital system is acceptable because tumors of this type represent a transitional epithelium carcinoma that affects the renal pelvis, renal ducts, bladder and urethra.

**Fig 4:**
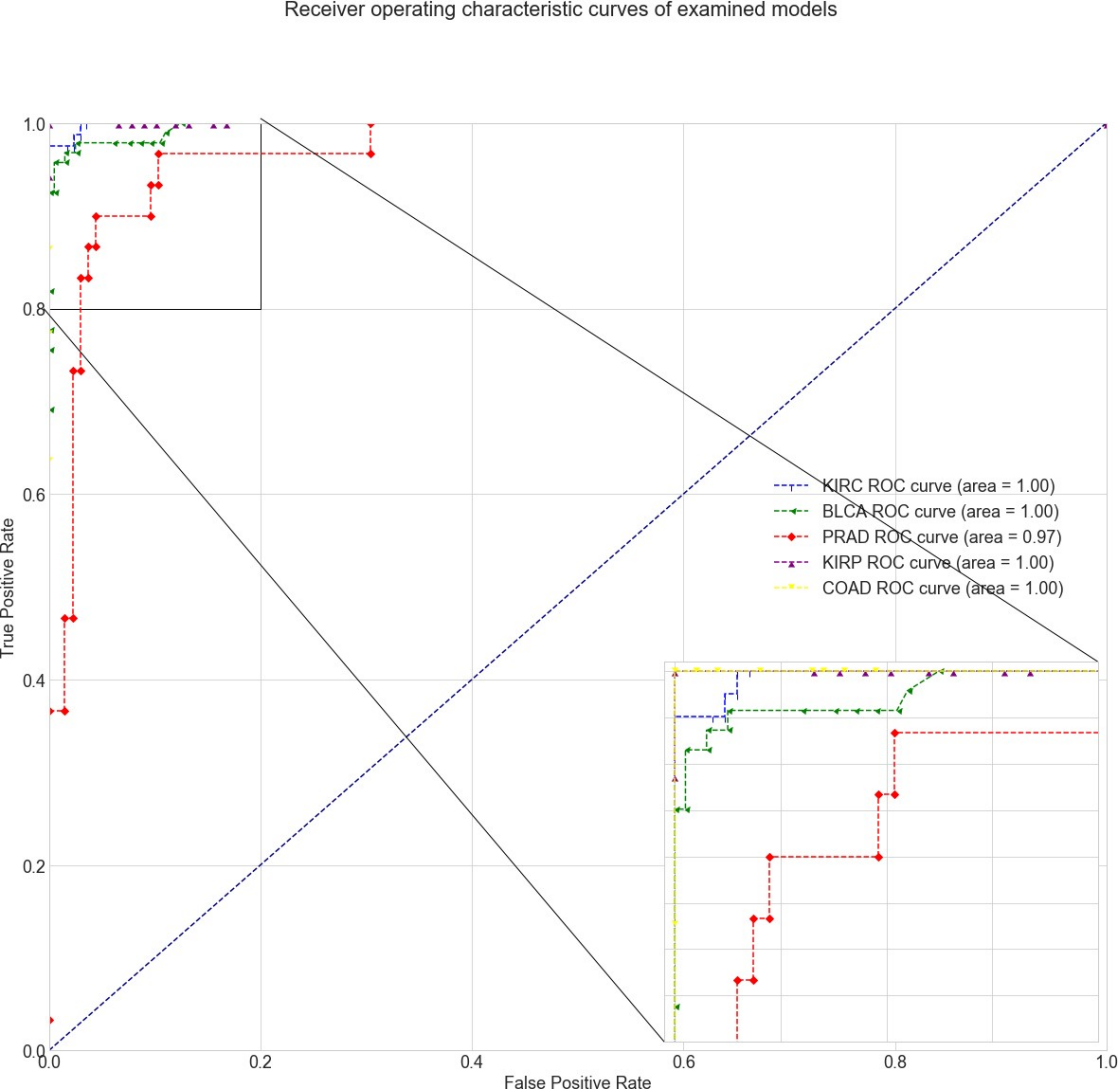
ROC curves for prostate adenocarcinoma (red), colon adenocarcinoma (yellow), urothelial bladder carcinoma (green), kidney renal papillary cell carcinoma (purple) and kidney renal clear cell carcinoma (blue) models. The lower-right inset shows a close-up of the upper-left parts of the AUC curves.

Classification efficacy of the resulting models was very high (Fig 4), and classification quality metrics, such as precision, recall, F1-score and the AUC for the test sets of the listed cancers, were higher than those for the prostate cancer model (Table 3). It should be noted that the resulting models included fewer sites (Table 4) and fewer correlated sites (S5–S8 Tables), indicating lower heterogeneity in these cancers compared to PC. The resulting models are specific and do not intersect with each other at the level of model and correlated sites, with the exception of *cg22274117* in the *ATXN1* gene (Kidney Carcinoma model). The analysis of these sites showed that they were previously reported as potential diagnostic biomarkers. The hypermethylation of the *ADHFE1* promoter in colorectal cancer has recently been demonstrated [64,65]. *CDH13* promoter methylation has been identified as a biomarker for bladder cancer [66]. Recently, *ENG* promoter hypermethylation was reported in several human cancers [67–69]. These results suggest that the LogLoss-BERAF framework could be effectively applied to different classification tasks.

## Conclusion

We have designed a framework for selection of a limited number of informative DNA methylation sites based on a combination of several feature selection methods and an ensemble-based classifier. We have applied the algorithm to the task of prostate cancer diagnostics and constructed a model with high classification efficacy metrics: 0.95 recall, 0.95 precision and 0.97 AUC. The method has also been demonstrated for methylation data from other types of cancers that are either co-localized with PC (colorectal cancer) or can be diagnosed using similar biological urine samples (bladder and kidney cancers), yielding model AUC values of 1.0. Based on the panel methylation pattern variability, a cluster of cancer samples was shown to have statistically significant higher recurrence rate. The resulting model has demonstrated robustness against input data errors, which can potentially allow the utilization of methylation level detection using other experimental strategies with lower resolution. The biological significance of the identified sites has been confirmed by previous studies.

## Supporting information

S1 Fig Methylation level distribution for 9 sites from PRAD diagnostic model constructed on the basis of the whole PRAD subset (Table 1). The dashed lines correspond to 95 and 5 quartiles, distribution medians are shown in red. Y axis shows methylation β-value.
(DOCX)

S1 Table Sample selection criteria for subsequent PRAD model construction. (XLSX)

S2 Table Methylation level values for the sites of the resulting PRAD model. (XLSX)

S3 Table Classification efficacy of tumor and non-tumor samples by PRAD gene and site methylation data analysis for different studies. (XLSX)

S4 Table PRAD model list of correlated sites. (XLSX)

S5 Table KIRC model list of correlated sites. (XLSX)

S6 Table KIRP model list of correlated sites. (XLSX)

S7 Table BLCA model list of correlated sites. (XLSX)

S8 Table CRCA model list of correlated sites. (XLSX)
