# Supplementary material for: LogLoss-BERAF: An ensemble-based machine learning model for constructing highly accurate diagnostic sets of methylation sites accounting for heterogeneity in prostate cancer
Source: PLoS One. 2018 Nov 2;13(11):e0204371. doi: 10.1371/journal.pone.0204371 (PMC6214495; doi:10.1371/journal.pone.0204371)
Supplement: S1 Fig — The dashed lines correspond to 95 and 5 quartiles, distribution medians are shown in red. Y axis shows methylation β-value. (DOCX) [file pone.0204371.s001.docx]

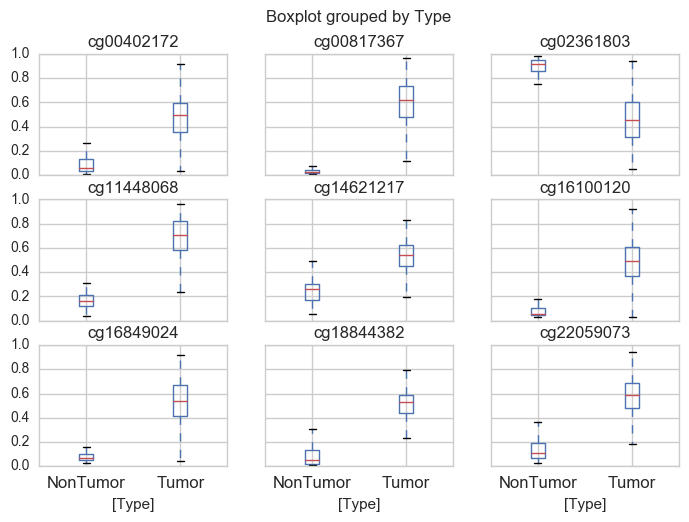


Supplementary Figure 1. Methylation level distribution for 9 sites from PRAD diagnostic model constructed on the basis of the whole PRAD subset (Table 1). The dashed lines correspond to 95 and 5 quartiles, distribution medians are shown in red. Y axis shows methylation β-value.
